# KAT2A changes the function of endometrial stromal cells via regulating the succinylation of ENO1

**DOI:** 10.1515/biol-2022-0785

**Published:** 2024-03-28

**Authors:** Kangkang Zeng, Hao Yin

**Affiliations:** Department of Obstetrics and Gynecology, Taihe Hospital, Hubei University of Medicine, 32 Renmin South Road, Maojian District, Shiyan 442000, Hubei, China

**Keywords:** endometriosis, embryonic stem cells, KAT2A, ENO1

## Abstract

Endometriosis is increasingly affecting women worldwide and research is focusing on identifying key targets in its pathogenesis. Changes in succinylation genes regulate the function of this protein and further influence the development of the disease. However, the role of succinylation genes in endometriosis is not clear from current studies. The expression of succinylation genes was determined in ectopic endometrium (EC) and ectopic patients with uterine fibroids (EN) by real-time quantitative PCR (qRT-PCR) and Western blot. Cell Counting Kit-8, transwell assays, and flow cytometry were used to assess endometrial stromal cells (ESCs) proliferation, apoptosis, migration, and invasion. KAT2A and ENO1 association was detected by qRT-PCR, immunofluorescence, and CoIP. We found that gene and protein levels of KAT2A were significantly increased in the EC group compared to EN group tissues. KAT2A silencing inhibited cell proliferation, migration, and invasion and promoted apoptosis. Western blot results showed that the expression of ENO1 and its succinylation was significantly upregulated in ECSc after KAT2A overexpression. CoIP results showed that KAT2A is positively bound to ENO1. Immunofluorescence also showed co-localized expression of KAT2A with ENO1. Furthermore, ENO1 overexpression reversed the effects of KAT2A silencing on the malignant behavior of ESCs. In summary, we found that succinylation of ENO1 mediated by KAT2A played a role in promoting the progression of endometriosis.

## Graphical abstract

**Figure:**
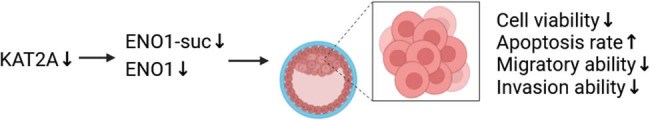

## 1 Introduction

Endometriosis is a classic chronic gynecological condition with a prevalence of about 10% of women of childbearing age [1]. People with endometriosis usually experience symptoms such as menstrual cramps, painful sexual intercourse, and infertility [2,3]. These symptoms seriously affect the quality of life of postmenopausal women. The characteristic of endometriosis is the abnormal localization of endometrial stromal cells (ESCs) and their ability to migrate and invade [4]. Therefore, to effectively alleviate the harmful progression of ESC, finding treatment targets for endometriosis remains a focus of research.

Current research indicates that alpha-enolase (ENO1) plays a key role in determining the characteristics of multiple RNA types [5]. ENO1 autoantibody is a new serum indicator for endometriosis [6]. It has been proven that endometrial cancer tissue has ENO1 mRNA levels. And the protein expression is higher than that of normal endometrial tissue in clinical samples, indicating that it may be used as a target for treating endometrial cancer [7]. And research has shown that patients expressing higher levels of ENO1 have shorter overall survival compared to those expressing lower levels. Therefore, controlling the expression of ENO1 may be an effective tool for treating patients with endometriosis. However, its mechanism is still unclear.

Succinylation is the procedure by which a succinyl group (co CH2-CH2-Co2h) is covalently bound to a lysine residue by a succinyl donor, for example, by enzymatic means [8,9,10]. More research on succinylation has shown that the level of succinylation is mainly controlled by the succinyl donors, succinyl-transferases and succinylases [11]. Succinate lysine acetyltransferase 2A (KAT2A) are members of the HAT family on chromosome 17q21.2 [12,13]. KAT2A also contains acetylated non histamines, such as CCAAT enhancer binding protein beta [14], Polarization kinase 4 [15], and T-box transcription factor 5 [16], and the loss of ribosome stability caused by the involvement of histamine glutamate KAT2A. It has also been proven to play an important role in the progression of cancer and many other cancers. However, there is no research confirming whether KAT2A can regulate ENO1 and reduce the condition of patients with endometriosis.

Therefore, in this study, we first examined the gene content of succinylation in ectopic patients with uterine fibroids (EN) and ectopic endometrial (EC) and further tested whether it could modulate ENO1 in the treatment of endometriosis.

## 2 Materials and methods

### 2.1 Tissue specimens

EN and EC tissues from patients with bile duct cancer in 2022 at Taihe Hospital were selected for this study. Patients all signed a relevant protocol prior to using clinical material. The research related to human use has been complied with all the relevant national regulations and institutional policies and in accordance with the tenets of the Helsinki Declaration, and has been approved by the Ethics Committee of Taihe Hospital.


**Informed consent:** Informed consent has been obtained from all individuals included in this study.
**Ethical approval:** The research related to human use has been complied with all the relevant national regulations, institutional policies and in accordance with the tenets of the Helsinki Declaration, and has been approved by the authors’ institutional review board or equivalent committee.

### 2.2 Cultivation and transfection of cells

ESCs were separated from the patients with endometriosis and cultured in Dulbecco’s Modified Eagle’s Medium (DMEM)/F12 (Gibco, NY, USA) at 37℃ and 5 % CO_2_ (the medium contained 15% FBS [Gibco, NY, USA] and 1 % P/S [Procell, China]). Once the cells were in a stable state, they were inoculated into six-well plates and continued to be cultured until the cell density reached 70%. KAT2A-targeting shRNA (sh-KAT2A) and negative control (shNC), KAT2A overexpressing vector (pcDNA3.1/KAT2A), and vector (pcDNA3.1) were purchased from General Biol (Anhui, China).

Cell transfection was performed using the Lipofectamine 2000 kit (Beyotime, China) according to the instructions. There is no need to add or change the culture medium after transfection, but the cells must be replaced with normal complete medium after 6 h of incubation, and the cells were used for further experiments.

### 2.3 Real-time quantitative PCR (RT-qPCR)

Cells were collected and lysed using TRIzol (9109, TaKaRa, Japan). Total RNA was extracted under liquid nitrogen freezing and then reverse transcribed to cDNA using PrimeScript™ RT Reagent Kit (RR036A, Takara, Japan). Finally, the target genes were subjected to fluorescent quantitative PCR using Power SYBR Green PCR Master Mix (4367659, Thermo, USA). The reaction conditions were: 50℃ for 3 min (×1 cycle), 95℃ for 3 min (×1 cycle), 95℃ for 10 s → 60℃ for 30 s (×40 cycle). The results were analyzed using 2^−△△Ct^ quantification method. GAPDH was used as an internal reference, and primers were prepared by the method of Shanghai Sangon Biotech (Table 1).

**Table 1:** The primer sequences for RT-PCR

Gene name	Forward primer (5′–3′)	Reverse primer (5′–3′)
SIRT5	TGGAGGAGGTTGACAGAGAGC	CTGCTGGGTACACCACAGA
SIRT7	ACGCCAAATACTTGGTCGTCT	AGCACTAACGCTTCTCCCTTT
KAT2A	CAGGGTGTGCTGAACTTTGTG	TCCAGTAGTTAAGGCAGAGCAA
KAT3B	GCTTCAGACAAGTCTTGGCAT	ACTACCAGATCGCAGCAATTC
CPT1A	ATCAATCGGACTCTGGAAACGG	TCAGGGAGTAGCGCATGGT
GAPDH	ACAACTTTGGTATCGTGGAAGG	GCCATCACGCCACAGTTTC

### 2.4 Analysis of the Cell Counting Kit-8 (CCK-8)

Cell viability was determined using the CCK-8 kit (C0038, Beyotime, China). Cells were inoculated in culture plates (96 wells, 1 × 10^4^/well) and incubated in an incubator (37°C, overnight). Cells were transfected separately the next day as previously described and incubated for 24 h with a change of solution after transfection. Then, CCK-8 solution (10 μL/well) was added and incubation was continued for 2 h (OD ≤ 2.0). Absorbance was measured using an enzyme marker and cell viability curves were plotted.

### 2.5 Apoptosis assays

After transfection, cells were collected in a flow tube using trypsin and centrifuged (1,000 rpm, 4 min) to remove the supernatant. The cells were resuspended in PBS and centrifuged. The resulting cells were resuspended using Annexin V-FITC conjugate (195 µL) and incubated with Annexin V-FITC (5 µL) and PI staining solution (10 µL) (25°C, 30 min according to the instructions of the Annexin V-FITC/PI Apoptosis Assay Kit [556420, BD, USA]). Apoptosis was analyzed using a flow cytometric analyzer (FACSCalibur, BD, USA).

### 2.6 Assay for the migration and invasion of cells

The migration and invasion behavior of cells was assessed by the conventional Transwell system. For cell migration assays, cells were digested, centrifuged, counted, and then diluted with serum-free medium at 5 × 10^5^/mL to make a cell suspension. Cell suspensions of 8 µm diameter were placed in sterile 24-well plates and added to the upper chamber of the cell suspension at 200 µL per well, while 500 µL of DMEM/F12 medium was added to the 24-well plates and incubated in a 37°C incubator for 48 h. After incubation for the required time, the cell chambers were removed, washed with sterile PBS, fixed in 4% paraformaldehyde (25℃, 20 min), and stained with crystal violet staining solution (25℃, 20 min). The cells were gently rotated in the upper chamber with a cotton swab to aspirate water and wipe away the inner side of the membrane. Record and photograph under an inverted microscope. For the cell invasion assay, the upper chamber of the transwell should be previously covered with a matrix gel (354234, Corning, USA), otherwise, the procedure is the same as for the cell migration assay.

### 2.7 Immunofluorescence

KAT2A rabbit antibody (ab153903, Abcam) and ENO1 mouse antibody (MA5-17627, Invitrogen) were used as primary antibodies according to the immunofluorescence method. Dnk pAb of rabbit IgG (Alexa Flavor 488) and Dnk pAb of goat IgG (Alexa Flavor 594) were used as secondary antibodies. Observations were made with a laser confocal microscope.

### 2.8 Co-immunoprecipitation (CoIP)

Add 1 mg of primary antibody against KAT2A or ENO1 to the extracted protein and incubate overnight at 4°C with slow shaking. The incubated material was co-incubated with 20 mL of protein A/G beads (4°C, 2 h). After the immunoprecipitation reaction, the IP product was centrifuged (1,500 rpm, 2 min, 4°C), the supernatant was aspirated off, and the beads were boiled for 5 min, followed by protein blotting to determine the expression of ENO1 and KAT2A as mentioned above.

### 2.9 Western blot

Total protein was extracted from the cells using RIPA lysate (P0013B, Beyotime, China) containing PMSF (ST506, Beyotime, China) and the protein concentration was determined using the BCA kit (20201ES76, Yisheng Biotechnology Co., Ltd., China). The primary antibody incubation was performed with the following antibodies: anti-SIRT5 (1:1,000, MA5-32464, Invitrogen), anti-SIRT7 (1:1,000, MA5-31904, Invitrogen), anti-KAT3B (1:1,000, MA1-16608, Invitrogen), anti-CPT1A (1:1,000, PA5-76788, Invitrogen), anti-KAT2A (1:1,000, ab153903, Abcam), anti-ENO1 (1:1,000, MA5-17627, Invitrogen), and anti-GAPDH (1:20,000, 60004-1, Proteintech, USA) were incubated overnight at 4°C, washed 3 times with TBST, and incubated with HRP-labeled secondary antibodies for 1 h. The ECL kits were used for the incubation of the membranes. ECL kit (P0018S, Beyotime, China) was used for chemiluminescence development. Using GAPDH as the internal reference, the gray value of each band was analyzed using the gel image analysis software ImageJ. The ratio of the gray value of the target protein to the internal reference protein band was calculated.

### 2.10 Statistical evaluation

The experimental data obtained are shown as mean ± standard deviation and were recorded, processed, and statistically analyzed using the software GraphPad Prism 6. The relationship between two and more groups of data was compared using *t*-test and one-way analysis of variance, respectively. *P* < 0.05 indicates a significant difference with statistical analysis.

## 3 Results

### 3.1 Tissue from patients with endometriosis showed significantly increased expression of KAT2A

**Figure 1:**
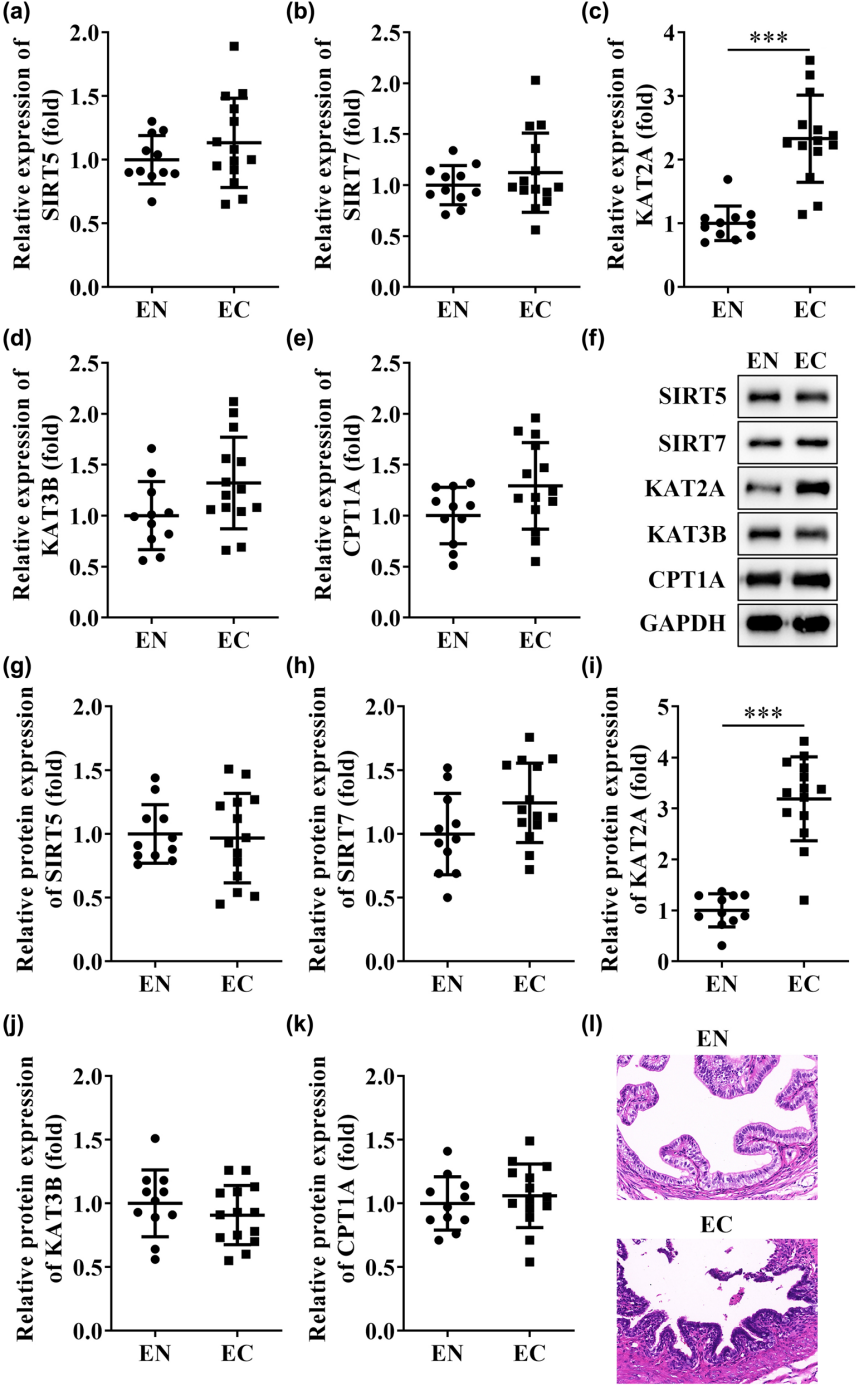
Expression of succinylases in tissues from patients with endometriosis. The mRNA expression of SIRT5 (a), SIRT7 (b), KAT2A (c), KAT3B (d), and CPT1A (e) in EN and EC tissues. (f) Western blot detected the expressions of SIRT5, SIRT7, KAT2A, KAT3B, and CPT1A. (g–k) Quantification of protein expressions of SIRT5, SIRT7, KAT2A, KAT3B, and CPT1A. (l) HE staining of EN and EC tissues. ***P < 0.001. Data are shown as mean ± standard deviation.

Succinylation is known to cause structural and functional alterations in related proteins during the development of many diseases. Among them, succinylases (KAT2A, KAT3B, CPT1A) and desuccinylases (SIRT5, SIRT7) are involved in the regulation. We therefore examined the genes for succinylation in EN and EC tissues. As seen in Figure 1, the PCR results showed no significant differences in the gene expression of SIRT5, SIRT7, KAT3B, and CPT1A between EC and EN groups (Figure 1a, b, d, e); however, KAT2A was significantly up-regulated in EC (Figure 1c). Similarly, the western blot results were consistent with the PCR, just KAT2A was significantly up-regulated in EC at protein level (Figure 1f–k). The hematoxylin eosin (HE) staining showed that compared with EN tissues, a large amount of inflammatory cell infiltration occurred in the EC group and organizational structure was damaged (Figure 1l).

### 3.2 KAT2A accelerated the malignancy of ESCs

**Figure 2:**
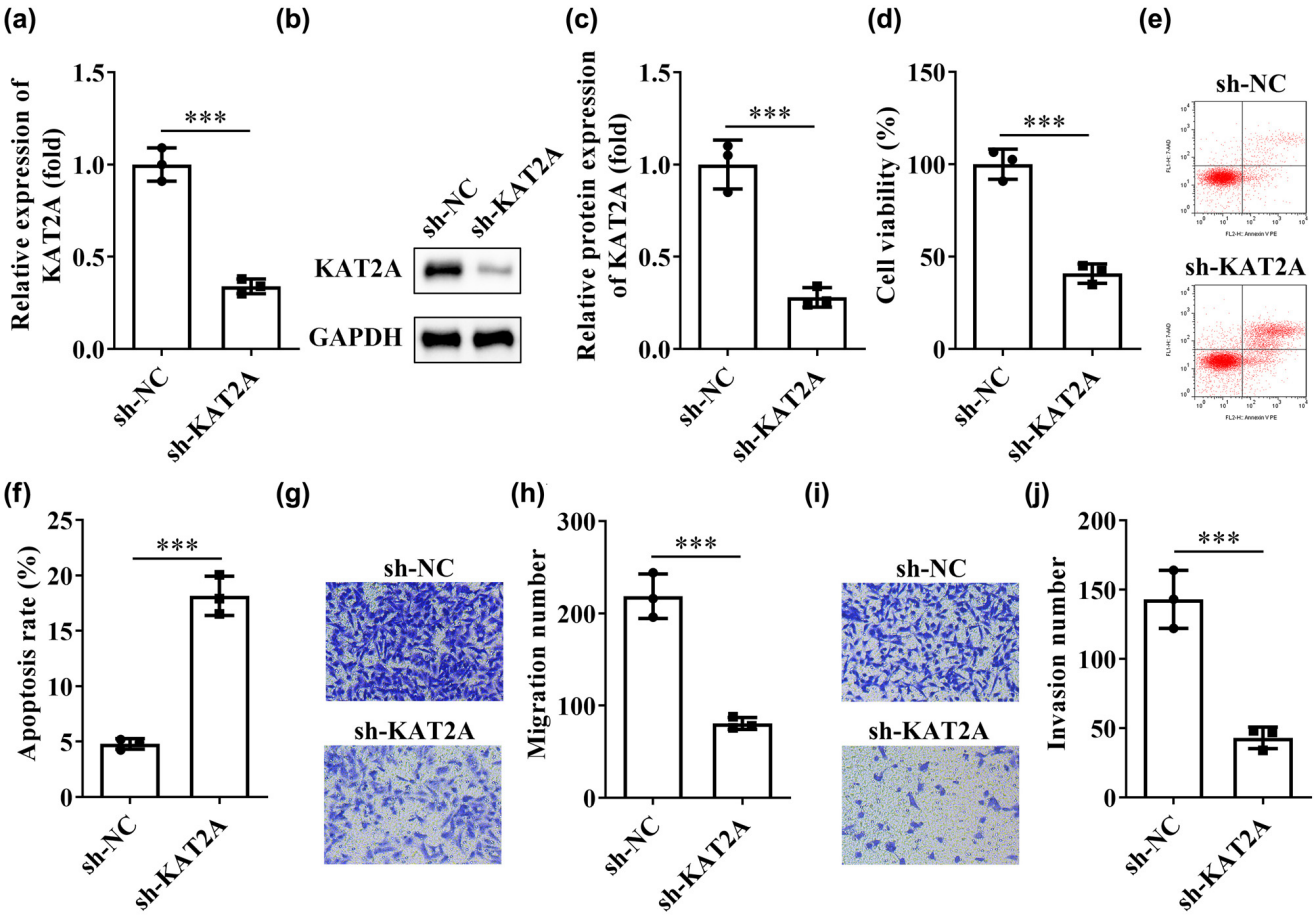
Effects of KAT2A on cell viability, cell apoptosis, migration, and invasion. (a) PCR and (b and c) WB assays for silencing efficiency of KAT2A. (d) The cell viability was analyzed. (e and f) Flow cytometry analysis shows the apoptotic rate. (g and h) The cell migration and (i and j) invasion were evaluated by transwell assay and respective statistics. ***P < 0.001. Data are shown as mean ± standard deviation.

We designed sh-KAT2A to silence KAT2A in ESCs, to test the biological effects of KAT2A. As shown in Figure 2a and b, PCR and WB results illustrated the successful silencing of KAT2A. The CCK-8 assay showed that the proliferative capacity of ESCs was significantly reduced in the sh-KAT2A group (Figure 2c). Flow cytometry results showed a significantly greater apoptosis of ESCs in sh-KAT2A group (Figure 2d and e). In addition, the invasion rate and migration rate of ESCs in the sh-KAT2A group were found to be significantly reduced by cell transwell assay (Figure 2f–i). All of the above results were performed with the sh-NC group as a control. Taken together, down-regulation of KAT2A significantly reduced the invasive and migratory ability of ESCs.

### 3.3 KAT2A could modulate the succinylation of ENO1

**Figure 3:**
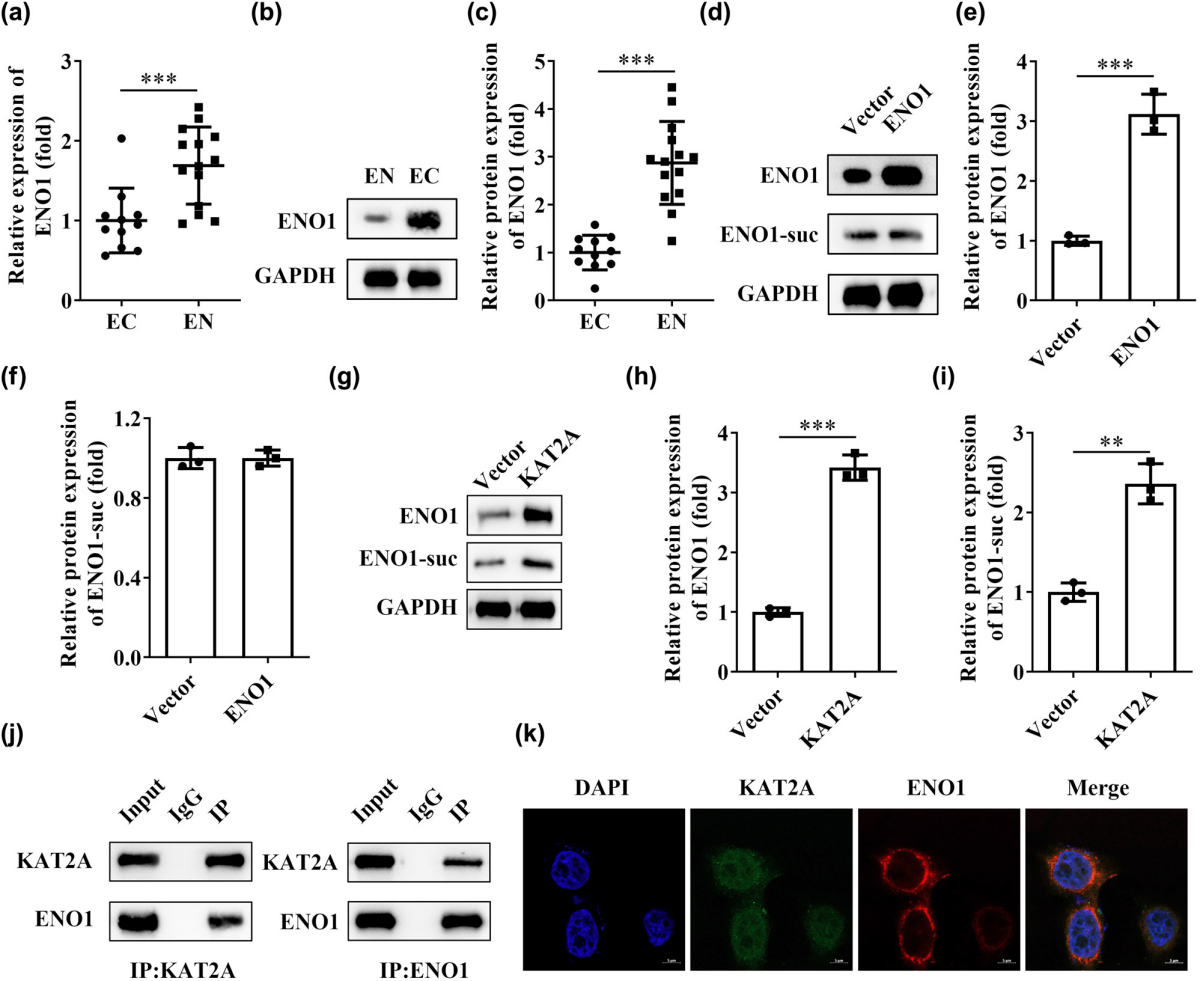
KATA2 could modulate the succinylation of ENO1. (a) The mRNA and (b) and (c) protein levels of ENO1 in EN and EC tissues. The ENO1 and ENO1-suc levels were detected by western blot after (d–f) ENO1 and (g–i) KAT2A overexpression. (j) CO-IP was used to detect the interaction of KAT2A with ENO1 protein. (k) Immunofluorescence double staining was used to detect the co-location of KAT2A and ENO1. **P < 0.01, ***P < 0.001. Data are shown as mean ± standard deviation.

It has been suggested that ENO1 could be a useful clinical marker for the diagnosis of endometriosis [6]. Therefore, we further tested whether KAT2A was involved in the regulation of ENO1 to reveal its biological mechanism in the development of endometriosis. As seen in Figure 3, the mRNA (Figure 3a) and protein (Figure 3b and c) levels of ENO1 significantly increased in the EN group. Then, we found after ENO1 overexpression, the ENO1 protein levels were significantly increased, while succinylation modification of ENO1 shows no difference (Figure 3d–f). Besides, the succinylation modification of ENO1 and ENO1 was significantly higher in the KAT2A overexpression group than in the control (Figure 3g–i). It was further found by CO-IP assay that KAT2A could positively react with ENO1 (Figure 3j). Subsequently, by immunofluorescence staining of KAT2A and ENO1, we found that KAT2A was mainly located in nucleus, and ENO1 was mainly located in cytoplasm (Figure 3k), which indicated that there was a reciprocal relationship between KAT2A and ENO1.

### 3.4 ENO1 reversed the effects of KAT2A on the apoptosis, migration, and invasion of ESCs

**Figure 4:**
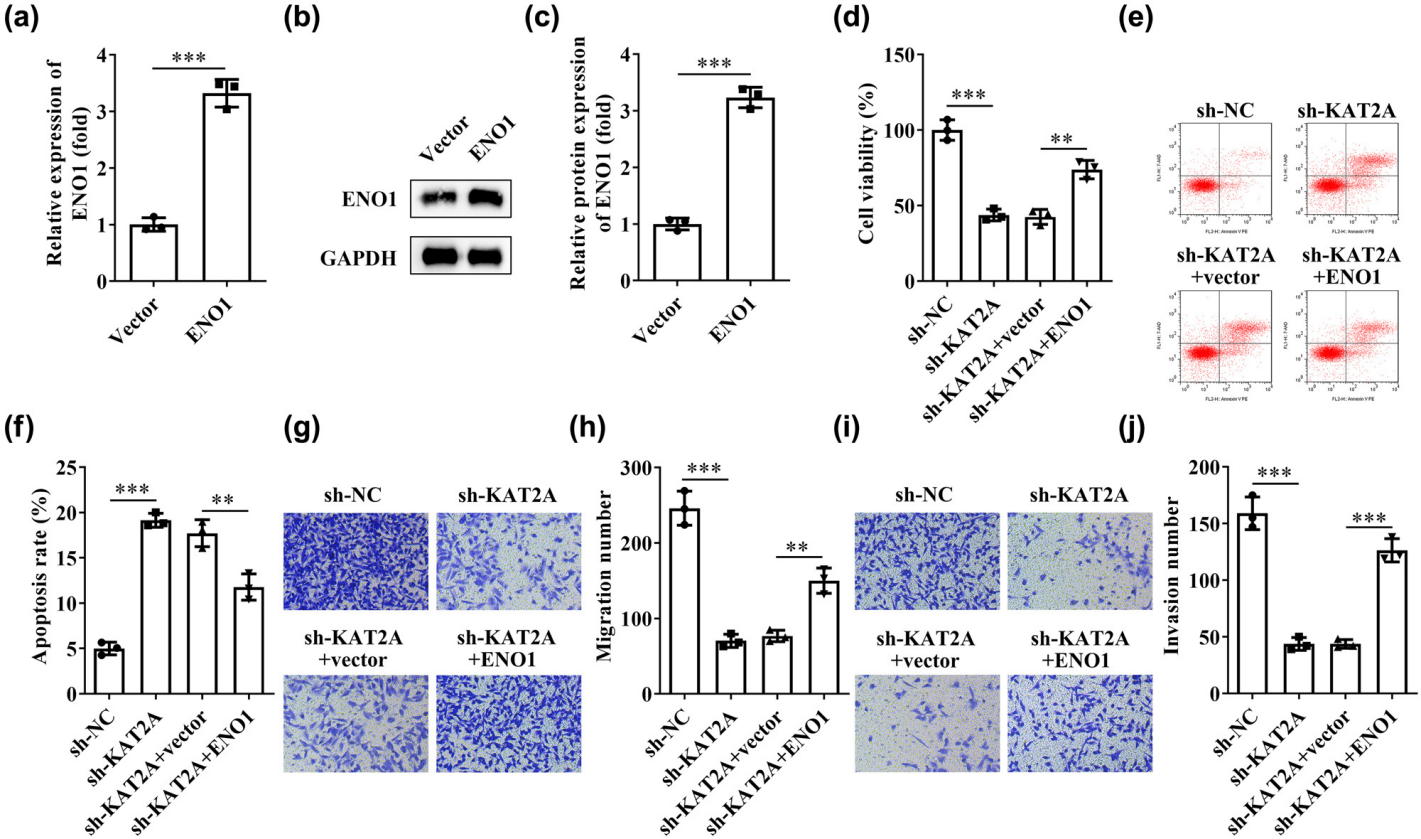
Effects of KAT2A and ENO1 on the progression of endometriosis (a) qPCR and (b and c) western blotting was used to evaluate the expression of ENO1 after transfection. (d) The cell viability was analyzed by CCK-8 assay in ESCs. (e and f) Flow cytometry analysis shows the apoptotic rate. The (g and h) cell migration and (i and j) invasion were evaluated by transwell assay and the respective statistical. **P < 0.01, ***P < 0.001. Data are shown as mean ± standard deviation.

To further validate the interaction between KAT2A and ENO1 in endometriosis progression, we transfected ESCs with sh-KAT2A and ENO1 overexpressing plasmid and tested the biological functions of ESCs. PCR (Figure 4a) and WB (Figure 4b and c) results indicated successful overexpression of ENO1. CCK-8 testing showed that the proliferation of ESCs in the sh-KAT2A group was significantly reduced compared to the sh-NC group, whereas the proliferation of ESCs was significantly enhanced following high expression of ENO1 trans (Figure 4d). By flow cytometry results, we found that for sh-KAT2A-induced ESCs cells, the apoptosis rate was significantly higher than that of sh-NC-induced cells, whereas after overexpression of ENO1 transfection, ESC apoptosis was significantly reduced (Figure 4e and f). In addition, both transwell assays illustrated that the invasive behavior and migratory capacity of ESCs were significantly lower in the sh-KAT2A group compared to the sh-NC group, but significantly higher after cotransfection with the ENO1 overexpression plasmid (Figure 4g–j). Overall, silencing of KAT2A increased apoptosis and inhibited the proliferation, migration, and invasion of ESCs; however, ENO1 reversed these results. These findings further suggested that KAT2A regulates the functions of ESCs via modulating ENO1 expression.

## 4 Discussion

The pathogenesis of endometriosis is complex and the treatment cycle is long, which leads to serious pathological changes in the endometrial tissue and affects daily life of patients [17,18,19]. Therefore, endometriosis is the main focus of research in obstetrics and gynecology. According to reports, ENO1 is an important biomarker for the development and prognosis of endometriosis [6]. In this study, we found an improvement in KAT2A expression in EN patients. Furthermore, KAT2A promoted the proliferation and metastasis of ECSs in vitro. Importantly, our research confirms that KAT2A can control the succinylation of ENO1, thereby reducing the harmful development of ESC.

Enolases are the important biologically active substances, also known as pyruvate dehydrogenase phosphatases, which convert both 2-phosphate-d-glycerate to phosphate-pyruvate (glycolysis) and the reverse conversion of phosphate-pyruvate to 2-phosphate-d-glycerate (glycogen synthesis) [20,21]. ENO1, one of the three enolase classes, is a glycolytic enzyme expressed in both the cytoplasm and the nucleus and plays an important function through its intracellular distribution. Research has shown that ENO1 performs higher in patients with endometriosis and is currently the most promising target drug in clinical practice [22,23]. Therefore, ENO1 targeting is crucial for exploring treatment methods for endometriosis. Protein post-translational modifications are reported to be dynamic, reversible protein modifications of types including phosphorylation, glycosylation, nitrosylation, methylation, acetylation, succinylation, etc., affecting almost all aspects of normal cell biology and pathogenesis [24,25,26]. Many studies have confirmed that modulating the methylation or acetylation of ENO1 can regulate the progression of a variety of pathologies [27,28,29,30,31,32]. However, no studies are showing that modulation of succinylation of ENO1 and thus regulation of endometriosis.

Succinylation modifications are a highly conserved class of modifications that play a key role in regulating the activity of a variety of proteins and in modulating a variety of signaling pathways. For example, succinylation of protein fibronectin 1 in ECM affects its degradation and thus tumor progression [33]. In addition, SIRT5 regulates the activity of ACOX1 to counteract oxidative stress and is expressed less in hepatocellular carcinoma [34]. In the present study, we confirmed the upregulation of succinate transferase KAT2A in endometriosis. *In vitro* experiments showed that KAT2A silencing inhibited the migration and invasion of ESCs, suggesting that KAT2A promotes the malignant development of endometriosis. Furthermore, overexpression of KAT2A significantly upregulated the levels of ENO1 and ENO1 succinylation, suggesting that KAT2A can promote the succinylation of ENO1. In addition, by fluorescence co-localization, we found that KAT2A and ENO1 were expressed in the nucleus and cytoplasm, respectively, and immunoprecipitation revealed that KAT2A could directly interact with ENO1. Meanwhile, ENO1 overexpression allowed KAT2A to inhibit the proliferation of ESCs and promote the malignant transformation of ESCs.

This was limited by having only one cell line used and lacking animal experiments. In our future studies, more cell lines will be used, and we will conduct animal experiments to further explore the specific mechanisms of KAT2A in endometriosis. Furthermore, it is still unclear how KAT2A interacts with ENO1 to function as a transcriptional agent, which is interesting and important. In addition, succinylation as a protein modification of lysine may affect ubiquitination degradation or phosphorylation, or affect protein modification at other sites. We will continue to investigate these issues.

## 5 Conclusion

In summary, we confirmed the high expression of KAT2A in endometriosis and its deteriorating effect on ESCs. Mechanistically, KAT2A interacted with ENO1 targeting and elevated the expression of ENO1 and its succinylated protein of ENO1. At the same time, ENO1 reversed the biological function of silencing KAT2A and promoted the deterioration of ESCs. This study provides new ideas for the diagnosis and management of endometriosis.
